# Bayesian Augmented Clinical Trials in TB Therapeutic Vaccination

**DOI:** 10.3389/fmedt.2021.719380

**Published:** 2021-10-22

**Authors:** Dimitrios Kiagias, Giulia Russo, Giuseppe Sgroi, Francesco Pappalardo, Miguel A. Juárez

**Affiliations:** ^1^School of Mathematics and Statistics, University of Sheffield, Sheffield, United Kingdom; ^2^Department of Drug Sciences, University of Catania, Catania, Italy; ^3^Department of Mathematics and Computer Science, University of Catania, Catania, Italy

**Keywords:** Bayesian hierarchical model, clinical trials, information sharing, *in silico* experiments, power prior, tuberculosis, therapeutic vaccine

## Abstract

We propose a Bayesian hierarchical method for combining *in silico* and *in vivo* data onto an augmented clinical trial with binary end points. The joint posterior distribution from the *in silico* experiment is treated as a prior, weighted by a measure of compatibility of the shared characteristics with the *in vivo* data. We also formalise the contribution and impact of *in silico* information in the augmented trial. We illustrate our approach to inference with *in silico* data from the UISS-TB simulator, a bespoke simulator of virtual patients with tuberculosis infection, and synthetic physical patients from a clinical trial.

## 1. Introduction

There will always be a push for innovative treatments for medical use—either drugs, devices, or therapies—in order to improve efficacy, cost-effectiveness, i.e., releasing new treatments suitable for public use requires stringent testing; hence, clinical trials must be closely regulated and scrutinised. Currently, this means the average duration of a clinical trial (Phase I–III) is 6–7 years, with a success rate of about 11% and mean cost of about USD 1.3 Billion (1–3). A promising avenue for improving on this issue is the use of formal models (mathematical and engineering) designed to simulate the effect of treatments in patients [see, e.g., (4, 5) and references therein]. A step further is using the information from *in silico* models to supplement clinical trials to decrease their size and duration, potentially speeding up the commercialisation of new interventions and reducing their cost to the public.

We concentrate on a novel therapeutic vaccination approach for treating tuberculosis (TB). Despite being treatable, TB was the cause of about 1.4M deaths in 2019 (6), the first cause of death by infectious pathogen. It is endemic in some South Asian countries, with a burden of about 40% (7). This is not a localised problem at all, and despite the low burden in the region, Europe has the highest number of new multi-drug resistant TB (MDR-TB) cases in the world^1^.

Despite continuous efforts, no new effective TB vaccines have been developed for almost a century, Andersen and Scriba (8); however, therapeutic vaccination is a promising treatment alternative (9–11). Within the STriTuVaD project^2^, we are developing methodology to generate *in silico* patients treated with RUTI as a coadjuvant in standard TB treatment (12), to supplement the data from a Phase II clinical trial. In this study, we propose a hierarchical Bayesian method, enabling the incorporation of the information from computer simulations onto an augmented *in silico* Phase II clinical trial investigating the efficacy of this therapeutic vaccination strategy.

## 2. Materials and Methods

One key complication in the development of new treatments for TB, with serious implications due to its impact on MDR-TB patients, is the rate of non-compliance (7, 13). From a clinical trial standpoint, the issue calls for increased recruitment in order to account for dropout rates, further increasing their cost. The use of *in silico* technologies has huge potential benefits, both clinical and financial, in these kinds of scenarios.

In section 2.1, we introduce the bespoke simulator of therapeutic RUTI vaccination of patients with TB in a Phase II clinical trial with a binary endpoint; section 2.2 describes the models used for the *in silico* and *in vivo* data sources, and section 2.3 details a model combining both sources of information to evaluate the end point of the clinical trial.

### 2.1. UISS-TB

The Universal Immune System Simulator (UISS) is a multi-scale, multi-organ, three-dimensional agent-based model (ABM) of the immune system, capable of simulating the dynamics of specific biological pathways at the molecular level. UISS has been successfully applied to numerous biological scenarios (14–17), with the majority of them including the simulation of vaccination evolution. A recent extension of the capabilities of UISS, the so-called UISS-TB, deals with the immune system at larger scales, enabling simulation of larger organs, such as the lungs, key in effective modelling the behaviour of TB.

Universal Immune System Simulator for Tuberculosis is a unique, bespoke ABM for the evolution of TB in the lung after RUTI vaccination (12, 18, 19), which can be considered as a computational model for RUTI vaccination. As such, under ASME V&V 40-2018 it is necessary to define its Context of Use under the Question of Interest [see, e.g., (20, 21), and references therein]. Based on the taxonomy proposed in Viceconti et al. (22), this process is carefully carried out in Curreli et al. (23) for using UISS-TB in augmented clinical trials. This ABM produces *in silico* data from a number of biological entities and chemical species (e.g., cytokines) for an individual virtual patient, identified and characterised through an initial vector of 22 features. In order to create cohorts of virtual patients, we use the novel approach from Juárez et al. (24), tailored for UISS-TB. In short, these features can be sampled, either at once or sequentially, and based on the joint distribution of the population characteristics, each virtual patient is then simulated using UISS-TB and the endpoint of the clinical trial recorded.

### 2.2. Modelling *in silico* and *in vivo* Data

Our main focus is to entertain models for the endpoint of the clinical trial with the aim of making them amenable to sharing information with the *in silico* data produced from the UISS-TB computer experiment. We propose a two layer approach: a layer dealing with the individual sources of information, either *in silico* or *in vivo* data, and a second combining the information in an augmented clinical trial.

For the former, we consider standard Bayesian (hierarchical) generalised linear models (hGLM), whereas for the latter we entertain a power prior approach based on the *in silico* model and a similarity measure, controlling the flow of information from the *in silico* experiment onto the augmented trial. We implement these Bayesian models using a benchmark prior, but our methodology can accommodate alternative prior specifications.

#### 2.2.1. Individual Sources of Information

For an individual source of information, either from *in silico* or *in vivo* data, we use a logistic hGLM. Given the stochastic nature of ABMs, our model (Equation 2) includes an additional source of uncertainty for the *in silico* data to capture the stochasticity from UISS-TB simulations.

Formally, for each group *j* = *R, C* —treatment (RUTI) and control—, each patient *i* = 1, …, *m* is identified by their corresponding vector of features, *x*_*i*_ = *x*_*i*1_, …, *x*_*ip*_, *p* = 22. We denote the *m* × *p* matrix of features by *X* = [*x*_1_, …, *x*_*m*_], and for each patient, we define


(1) $$\begin{array}{l} r_{i}^{j}=\{\begin{array}{ll} 1 & i\text{-th patient in group }j\text{ has a negative sputum culture}\\ 0 & \text{otherwise} \end{array}, \end{array}$$

with $P\left(r_{i}^{j}=1\right)=\theta_{i}^{j}$ the individual probability of a negative sputum smear count. To control for individual characteristics, we assume


(2) $$\begin{array}{l} g\left(\theta_{i}^{j}\right)=\log\frac{\theta_{i}^{j}}{1-\theta_{i}^{j}}=\{\begin{array}{ll} \mu^{j}+u_{i}^{j}+x_{i}^{j}\beta & \text{for }in\;silico\text{ data}\\ \mu^{j}+x_{i}^{j}\beta & \text{for }in\;vivo\text{ data} \end{array}, \end{array}$$

where **β** the vector of coefficients adjusting for individual features, μ^*j*^ is related to the baseline rate of conversion, and $u_{i}^{j}$ is the random effect accounting for the variability arising from simulating a specific profile *i* on UISS-TB, with $u_{i}^{j}∽\text{N}\left(u_{i}^{j}|0,{\sigma_{i}^{j}}^{2}\right)$ and $\sigma_{i}^{j}$ elicited from repeated measurements of the corresponding profile. This formulation is readily simplified if the underlying simulator is mechanistic instead, by letting $\sigma_{i}^{j}=0$.

We can then enable evaluation of the endpoint using both arms, by realising that log-odds ratio,


$$\begin{array}{l} g\left(\theta_{i}^{R}\right)-g\left(\theta_{i}^{C}\right)=\log\left(\frac{\theta_{i}^{R}}{1-\theta_{i}^{R}}/\frac{\theta_{i}^{C}}{1-\theta_{i}^{C}}\right)=\left(\mu^{R}-\mu^{C}\right)+\left(x_{i}^{R}-x_{i}^{C}\right)\beta , \end{array}$$

is the expected log-difference in performance, adjusted for individual characteristics. Thus,


(3) $$\begin{array}{l} \delta =\exp\left[\mu^{R}-\mu^{C}\right], \end{array}$$

is the endpoint of the trial, our quantity of interest.

#### 2.2.2. Prior Setup

For benchmarking, we propose a conventional Gaussian-Gamma prior,


(4) $$\begin{array}{l} \pi \left(\mu^{j},\beta ,\omega \right)\propto \text{N}\left(\mu^{j}|m^{j},{\tau^{j}}^{-1}\right){\text{N}}_{\text{p}}\left(\beta |\eta ,\omega^{-1}I_{p}\right)\text{ Ga}\left(\omega |c_{\omega },d_{\omega }\right), \end{array}$$

with the parameters $\left\{m^{j},\tau^{j},c_{\omega },d_{\omega }\right\}$ fixed to reflect relative little prior information and use π^*^η ∝ 1.

### 2.3. Combining Information From *in silico* and *in vivo* Data

In order to enhance the precision in evaluation of the endpoint and reduce the number of real patients needed, we want to combine the information from fitting (Equation 2) to the *in silico* UISS-TB data with the *in vivo* data. From our Bayesian approach, it is natural that the posterior distribution from the *in silico* data to function as the prior for the *in vivo* data model, with an additional parameter, the so-called power prior, for controlling the amount of information from the *in silico* data going into the combined model as in Haddad et al. (25). The power parameter is set based on a measure of compatibility between the *in silico* to the *in vivo* data, by using a weight function to account for dissimilarities in the distributions, and thus controlling the impact the virtual cohort has on the combined model.

It is worth noting here that, in practise, there will be a subset of features common to both *in silico* and *in vivo* experiments, susceptible of information sharing, whereas a number of features will be unique to each experiment. Formally, let **β**_*s*_ be the vector of coefficients associated with the *in silico* data model and **β**_*v*_ to the physical data model. Then, **β**_*c*_ = **β**_*s*_ ∩ **β**_*v*_ represents the common parameters to both experiments, and **β**_*v*−*c*_ the parameters from the *in vivo* model only, so that β_*v*_ = {β_*v*−*c*_, **β**_*c*_}. The likelihood from the *in silico* model can be expressed as,


(5) $$\begin{array}{l} \text{L}\left(\beta_{s}\;;\;D_{s}\right)\propto f\left(r|\beta_{s},X\right)=\overset{m}{\underset{i=1}{\prod }}f\left(r_{i}|\beta_{s},x_{i}\right), \end{array}$$

with *D*_*s*_ the *in silico* data. Hence, the posterior distribution of **β**_*s*_ is


(6) $$\begin{array}{l} \pi \left(\beta_{s}|D_{s}\right)\propto f\left(r|\beta_{s},X\right)\;\pi \left(\beta_{s},\eta ,\Sigma ,\omega ,\mu .\lambda \right), \end{array}$$

Using similar notation, we denote L(**β**_*v*_ ; D_*v*_) the likelihood from the physical clinical trial, with D_*v*_ the *in vivo* data, with


(7) $$\begin{array}{l} \pi \left(\beta_{v}|D_{s},D_{v}\right)\propto \text{L}\left(\beta_{v}\;;\;D_{v}\right)\;\pi \left(\beta_{c}|D_{s}\right)\;\pi \left(\beta_{v-c},\eta ,\Sigma ,\omega ,\mu ,\lambda \right), \end{array}$$

the posterior distribution of **β**_*v*_, where π(**β**_*c*_ | D_*s*_) is the joint marginal posterior of the common feature coefficients from the *in silico* data and


(8) $$\begin{array}{l} \pi \left(\beta_{c}|D_{s}\right)=\int \pi \left(\beta_{s}|D_{s}\right)\text{d}\beta_{s-c}, \end{array}$$

with **β**_*s*−*c*_ the set of parameters from the *in silico* model only.

Summarising, our model in Equation (7) takes into account the information coming only for the *in vivo* model, i.e., L(**β**_*v*_ ; D_*v*_), and draws all prior information for the common parameters to both experiments based on the joint marginal posterior distribution from *in silico* in Equation (8).

#### 2.3.1. Measure of Similarity

As it stands, π(**β**_*v*_ | D_*s*_, *D*_*v*_) takes the information coming from the *in silico* model at face value, i.e., the same weight is assigned to the information from the computer experiment and the clinical trial. In order to have a mechanism to control the amount of information shared, we consider a power prior approach (26–28), in which the prior distribution in Equation (8) is weighted based on a measure of compatibility α, with 0 < α < 1, and, therefore, the posterior distribution of **β**_*v*_ in Equation (7) updates in,


(9) $$\begin{array}{l} \pi \left(\beta_{v}|D_{s},D_{v}\right)\propto \text{L}\left(\beta_{v}\;;\;D_{v}\right)\;\pi {\left(\beta_{c}|D_{s}\right)}^{\alpha }\;\pi \left(\beta_{v-c}\right). \end{array}$$

**Figure 1:**
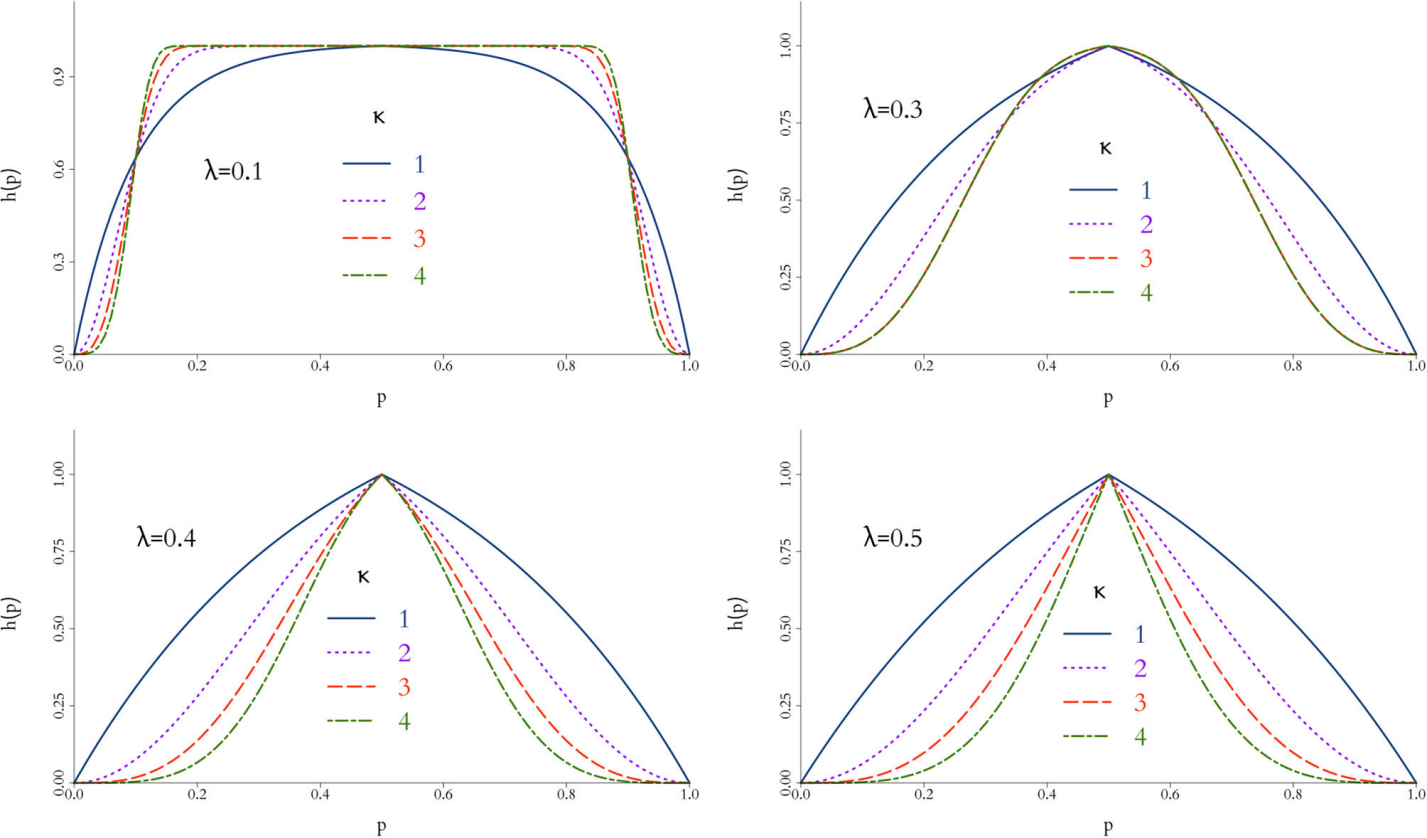
Behaviour of weight function *h*(*p*) in Equation 10 for different values of λ and κ. The closer *h*(*p*) to 1, the more compatible the simulations are to the physical data and a larger virtual cohort is used in the combined trial. In general, *h*(*p*) is rather insensitive for small values of λ and more discriminatory as κ increases.

We follow Haddad et al. (25) and express α = *m*/*M*, with *M* the size of the virtual patient cohort and 0 < *m* < *M* the effective size of the *in silico* trial. To provide a measure of agreement, let π(δ_*s*_ | D_*s*_) and π(δ_*v*_ | D_*v*_) be the posterior distribution of the endpoint from the virtual and the physical cohorts, using the conventional non-informative prior distribution. One would expect *p* = P(δ_*v*_ < ϕ_*s*_) to be close to 0 or 1 if the virtual cohort provided dissimilar information to the physical; thus, *p* can be treated as a measure of agreement. We use a weight function, *m* = *h*(*p*) × *m*_max_, based on *p*, in such a way that *m* → 0 if *p* → 0, 1 and *m* → *m*_max_ if *p* → 1/2, with *m*_max_ the maximum number of virtual patients allowed in the combined trial. Formally, we consider the symmetric weight function around 1/2,


(10) $$\begin{array}{l} h\left(p\;;\;\lambda ,\kappa \right)\propto \{\begin{array}{ll} 1-\exp\left[-{\left(\frac{p}{\lambda }\right)}^{\kappa }\right] & p<0.5\\ 1-\exp\left[-{\left(\frac{1-p}{\lambda }\right)}^{\kappa }\right] & p\geq 0.5 \end{array}, \end{array}$$

where the parameters λ and κ control the scale and shape of the function, respectively, and hence the stringency of the penalty as *p* deviates from 1/2. In short, larger values of λ and/or κ provide a faster decrease from the peak of the function, therefore decreasing the effective size *m*; this behaviour is illustrated in Figure 1 for different combinations of {λ, κ}.

## 3. Results

To illustrate our methodology, we simulate a two arm Phase IIb trail to test the efficacy of RUTI in drug-sensitive patients with TB. Both groups start with standard MDR-TB treatment from day 0; additionally, the intervention group is injected with 25 μg RUTI after at day 7. Efficacy is measured as percentage of patients with sputum culture negative at day 15. There are 150 patients in the *in vivo* cohort, split equally at random to each arm. We generated 150 profiles as in Juárez et al. (24), 75 per arm, and ran 100 UISS-TB simulations per virtual patient. Table 1 displays summary statistics from each group.

**Table 1:** Key patient features.

**Group**	**Feature**	**Range**	**n**	**Min**	***q*_0.025_**	***q*_0.5_**	***q*_0.975_**	**Max**
*In vivo* Ctrl	Age	[10, 80]	75	32	34.85	44	52.45	56
	Sputum	[0, 10, 000]		4,879	4,896	5,010	5118.3	5,149
	BMI	[18.5, 35]		20.68	22.58	27.21	31.01	32.16
	INF_γ_	[0, 268.2]		13.56	13.89	25.13	32.09	33.23
*In vivo* RUTI	Age	[10, 80]	75	33	35.7	44	54	55
	Sputum	[0, 10, 000]		4,865	4922.2	5,004	5,100	5,144
	BMI	[18.5, 35]		22.03	23.49	26.87	32.03	33.88
	INF_γ_	[0, 268.2]		14.11	16.41	24.75	32.48	36.86
*In silico* Ctrl	Age	[10, 80]	75	32	34.55	45	53.15	62
	Sputum	[0, 10, 000]		4,871	4890.85	5,006	5,082	5133
	BMI	[18.5, 35]		21.18	22.91	26.58	30.70	30.95
	INF_γ_	[0, 268.2]		15.3	15.88	24.33	30.77	33.21
*In silico* RUTI	Age	[10, 80]	75	35	36.85	44	51	53
	Sputum	[0, 10, 000]		4,892	4910.7	5,002	5097.35	5,178
	BMI	[18.5, 35]		19.87	22.02	26.42	30.04	31.85
	INF_γ_	[0, 268.2]		13.47	18.42	24.94	34.62	36.52

*Admissible ranges of UISS-TB features [see (24)] and sample summary statistics (min, max, 2.5, 50, and 97.5% quantiles) from each subgroup in the virtual and physical cohorts used in the augmented Phase IIb clinical trail of RUTI*.

**Figure 2:**
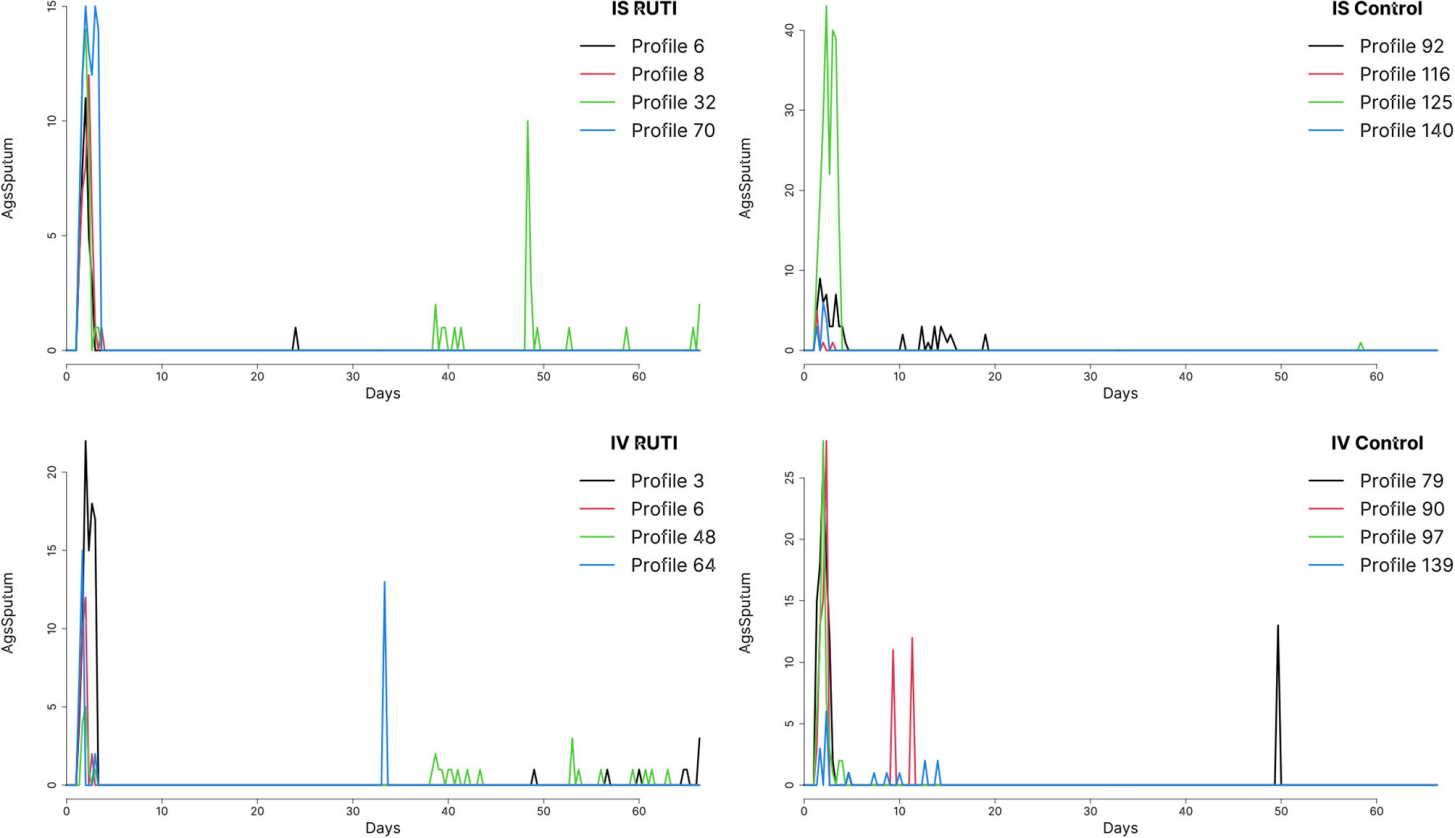
Sputum levels in time from profiles chosen at random from both RUTI and Control groups in the *in vivo* (IV) and *in silico* (IS) cohorts, using run 49 out of a 100 available.

In Figure 2, we plot the time series of sputum culture for arbitrary chosen profiles from a single run of the UISS-TB simulations, also selected at random. Patients in both RUTI and Control groups appear to have high values of sputum culture at the beginning of the trial —days 0 to 7— and seem to show different behaviour at the later stages of the trial, with small bumps at isolated days, specifically the control group in the *in vivo* cohort. The majority of patients seem to return to a state of negative sputum culture after day 14 on average.

In order to obtain a reasonable estimate for each unique profile, we average the sputum culture over the 100 UISS-TB simulations and set a common threshold of 0.05 to obtain $r_{i}^{j}$ in Equation (1) for each patient *i* = 1, …, 75 and each group *j* = R, C, where 1 indicates a patient with sputum culture negative and 0 otherwise. The reason for doing so is the variability coming from replicates of a specific profile using UISS-TB, summarised into the random effect term in our model in Equation (2) and analysed in section 3.1.

### 3.1. Random Effect in the *in silico* Cohort

In order to measure the simulation uncertainty in the UISS-TB output, i.e., the variability of the output from a patient with the same profile, we first fit (Equation 2) without $u_{i}^{j}$ to both groups using all 75 patients with unique profiles and their 100 runs as individual patients. We then compare the resulting posterior distribution of the odds with the one from using a flat prior for the precision of the random effect.

**Figure 3:**
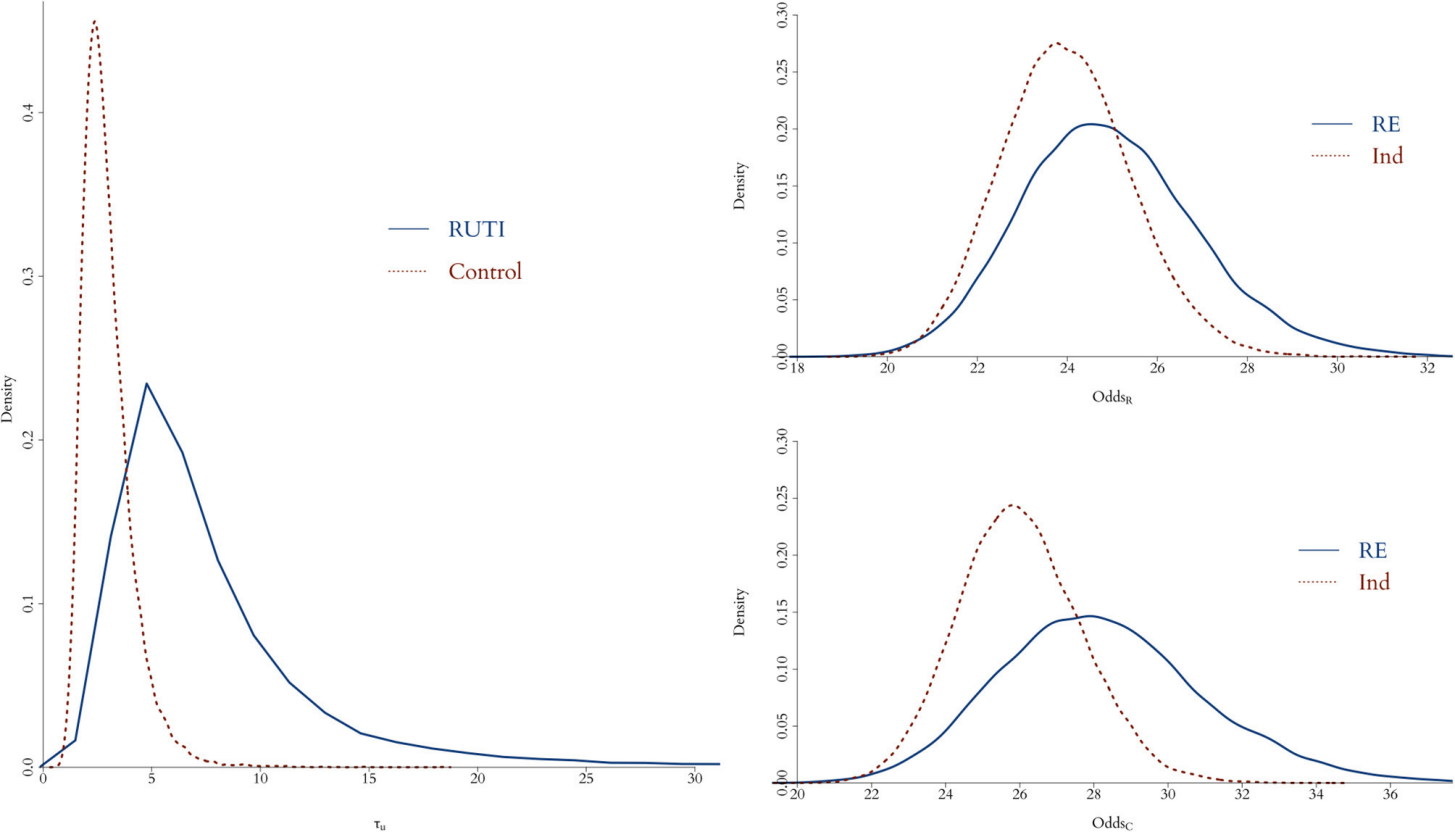
Posterior distribution of odds for both RUTI (top left) and Control (bottom left) groups, when either using profiles with identical initial vector of features as individual patients (red dotted) or taking each profile with its corresponding 100 simulations as a replicate of a specific patient (solid blue). On the left panel, the posterior distributions of the random effect precision from each group is displayed.

**Figure 4:**
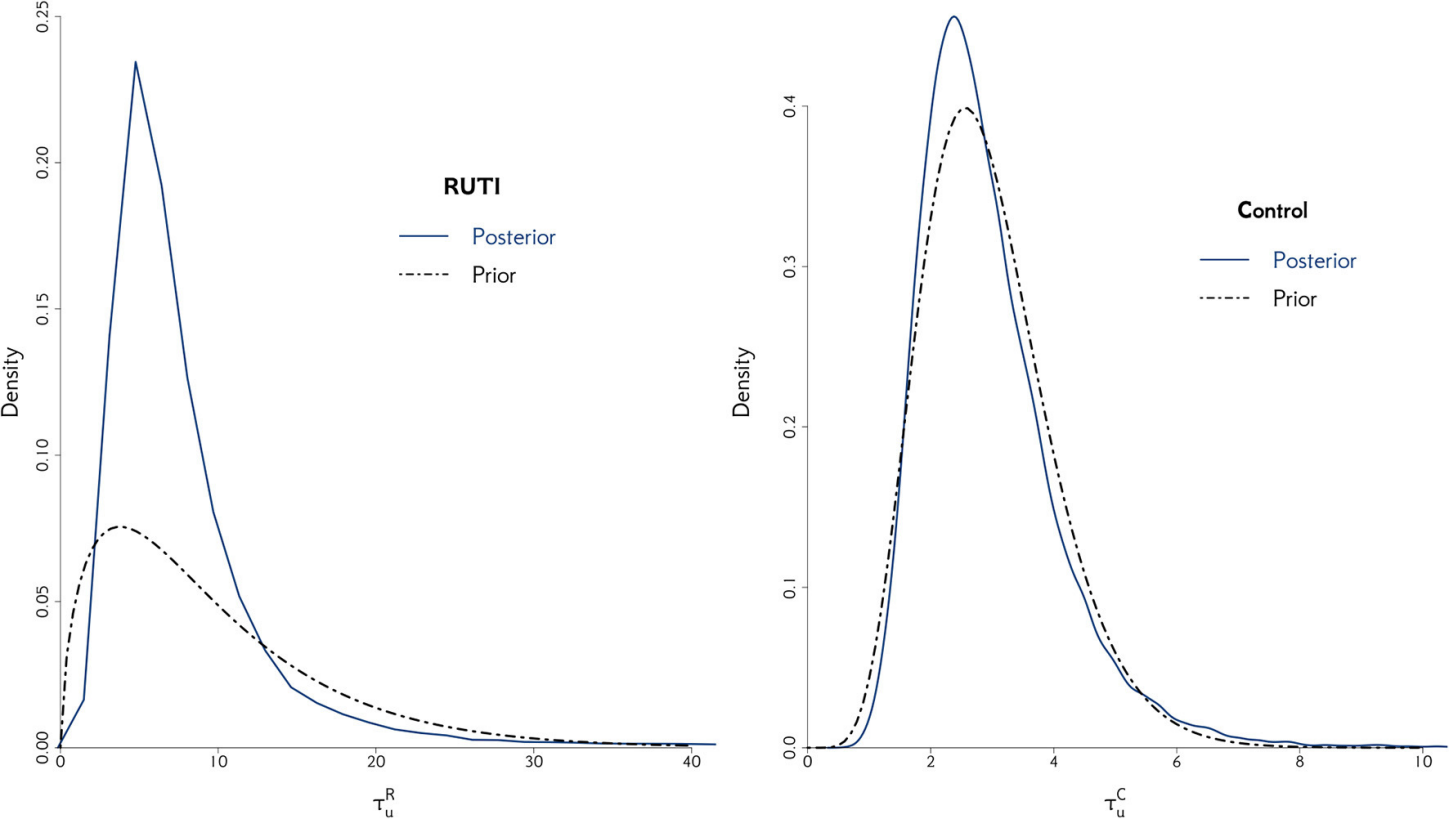
The posterior distribution of the random effect precision is displayed for both RUTI and Control groups, along with their corresponding best gamma distribution fit. In both cases, we elicit the prior distribution for τ_*u*_*i*__ from the combined models.

As illustrated in Figure 3, when the replicates of each profile are treated as individual patients, the posterior distribution of the odds is slightly different compared to the case where we take into account the fact that we do know replicates exist. Overall, the interpretation of the fitting is fundamentally the same; in both RUTI and Control groups, the odds are similar (rightmost panels). However, in the former case there is a clear source of variability missing, even if it is small, evidenced by the posterior distribution of the precision of random effect, $\tau_{u_{i}}=1/\sigma_{u_{i}}^{2}$, on the leftmost panel. Hence, we use the model with a random effect for the *in silico* data, eliciting a Gamma prior distribution for τ_*u*_*i*__, depicted in Figure 4.

### 3.2. Determining the Contribution of the *in silico* Experiment

**Figure 5:**
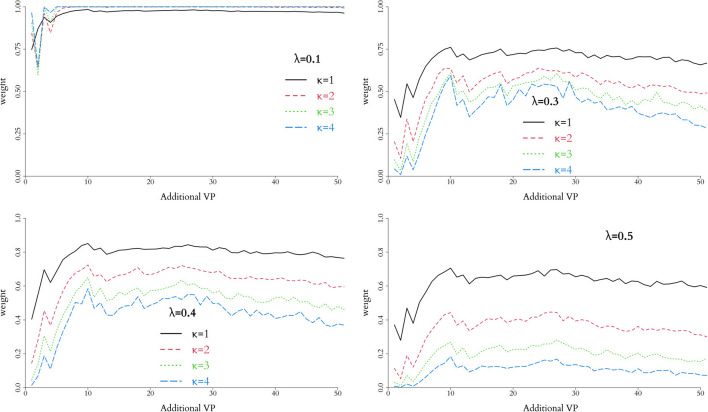
Weight associated with each combination of parameters in the weight function, *h*(*p*), for the RUTI group. Relative low values of λ do not provide clear separation, regardless of the potential number of additional virtual patients. For the data observed, λ = 0.4 and κ = 1 provide appropriate separation and suggest adding 12–30 virtual patients to the combined trial.

**Figure 6:**
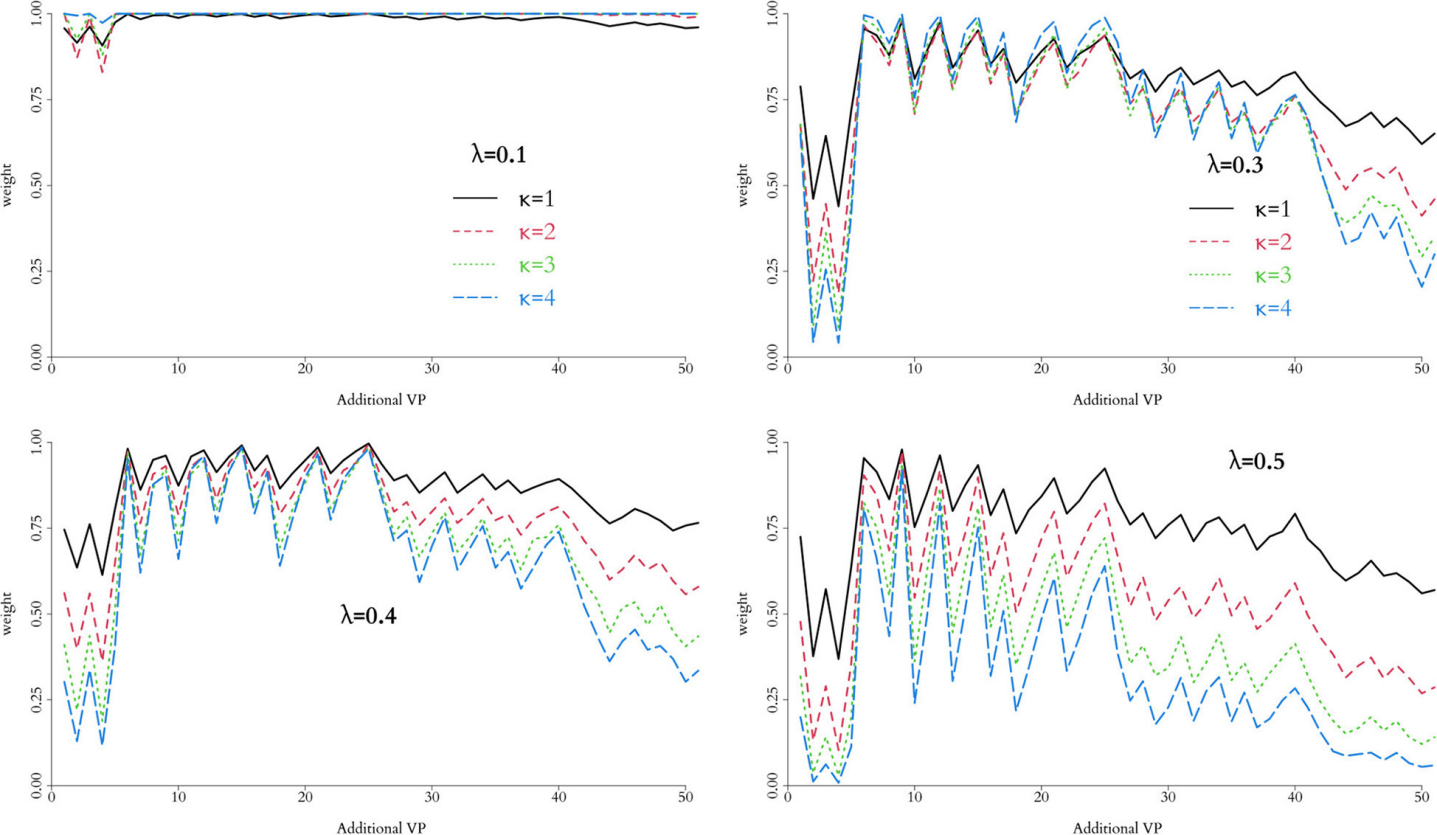
Weight associated with each combination of parameters in the weight function, *h*(*p*), for the Control group. Relative low values of λ do not provide clear separation, regardless of the potential number of additional virtual patients. For the data observed, λ = 0.4 and κ = 1 provide a reasonable separation and suggest adding 18-25 virtual patients to the combined trial.

In order to control the supplemental amount of information from the *in silico* data into the augmented trial, we perform preliminary runs adding one *in silico* patient at a time and analyse the behaviour of the weight, α. Ideally, we seek for the point where α is higher, while controlling the discrepancy measured by Equation (10) for different combinations of λ and κ. The behaviour of this penalty function is illustrated in Figure 1, and the resulting weight from adding single *in silico* patients sequentially to the RUTI and Control cohorts presented in Figures 5, 6, respectively.

As expected, low values of λ, regardless of κ, yield weights unable to discriminate the information contribution from the *in silico* experiment, particularly for small numbers of added patients. Larger values, in contrast, allow for better discrimination, which can be fine tuned by varying κ. In our implementation, we use λ = 0.4 and κ = 1 enabling identification of an optimal number of added virtual patients, i.e., a value of α that keeps the similarity of the *in silico* and *in vivo* cohorts at an acceptable level, allowing us to increase the sample size of the augmented clinical trial potentially improving the precision in estimation of the end point.

For the RUTI group, the optimal number of added patients appears to be between 12 and 30, whereas for the Control group between 18 and 25. Therefore, for our implementation we will use a batch of $n_{s}^{\text{R}}=26$ and $n_{s}^{C}=25$ *in silico* patients to combine with the *n*_*v*_ = 75 *in vivo* patients in the augmented clinical trial. Table 2 summarised some of the key characteristics of the posterior distribution of the odds from both groups with batches of various sizes added. Results from the Control group change ever so slightly with the number of the added virtual patients. In the Control group, adding more than approximately 15 patients and up to 35 seems to keep a similar posterior mean and slightly smaller interquartile range (IQR), whereas, in the RUTI group, adding virtual patients results in a small downward shift on the posterior mean with similar ranges.

**Table 2:** Summary statistics (2.5, 50, and 97.5% quantiles) and interquartile range (IQR) of posterior distributions of the odds from each group after adding virtual patient batches of different sizes (*n*_*s*_) to the *in vivo* cohort (*n*_*v*_).

**Group**	***n*_*v*_**	***n*_*s*_**	***q*_0.025_**	***q*_0.5_**	***q*_0.975_**	**IQR**
Control	75	0	0.326	0.538	0.875	0.182
		5	0.301	0.512	0.845	0.182
		15	0.334	0.542	0.875	0.181
		25	0.333	0.544	0.873	0.181
		35	0.328	0.541	0.873	0.182
		45	0.322	0.533	0.860	0.181
RUTI	75	0	0.383	0.649	1.081	0.229
		5	0.340	0.607	1.057	0.236
		15	0.360	0.629	1.067	0.230
		25	0.367	0.630	1.063	0.230
		35	0.366	0.627	1.057	0.230
		45	0.361	0.628	1.060	0.232

### 3.3. An Augmented Clinical Trial

**Figure 7:**
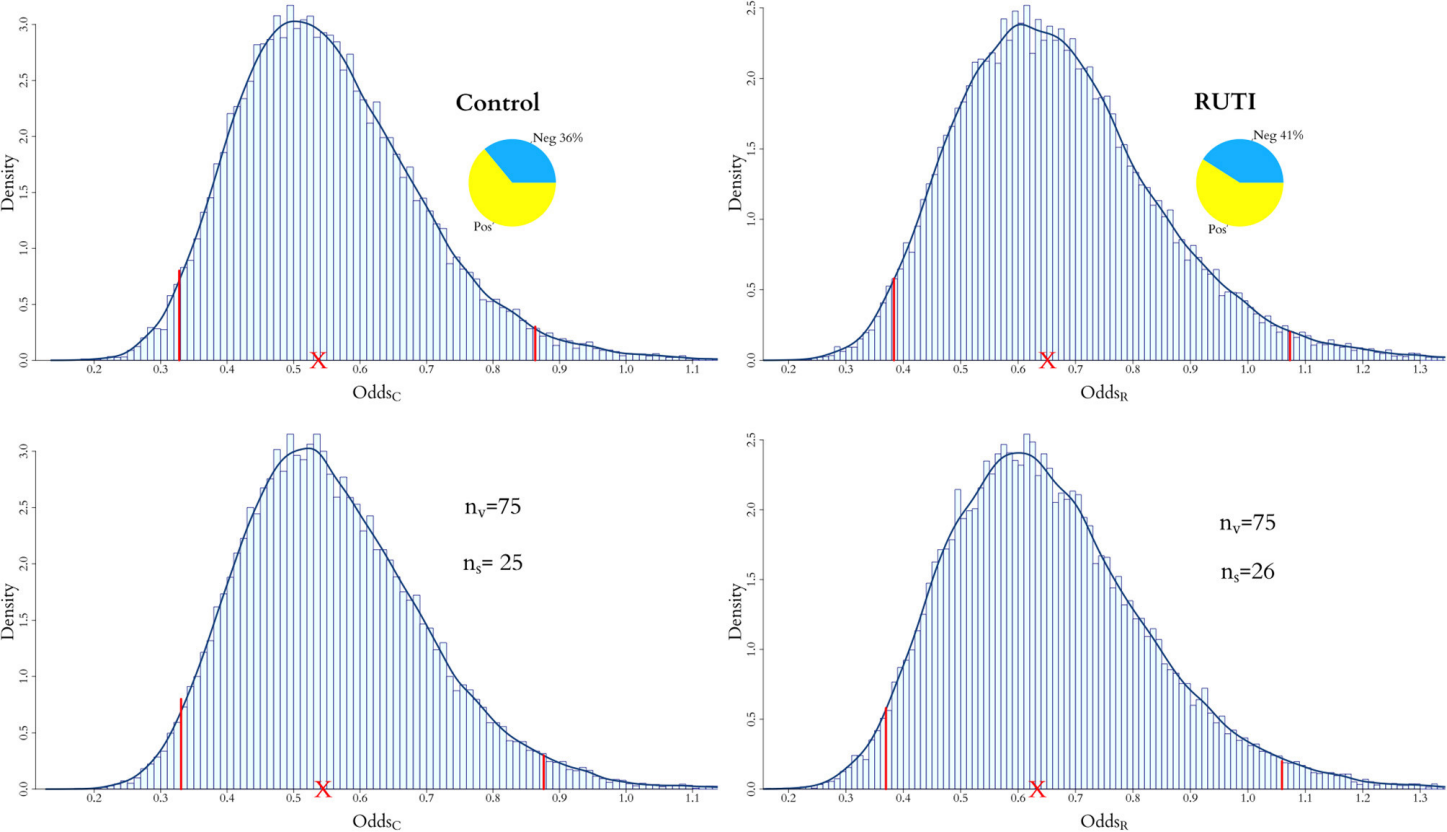
Posterior distribution of odds for both RUTI and Control groups, when only the *in vivo* cohort is fitted and when both the *in vivo* and *in silico* cohorts are combined for the full model. For the latter, the optimal number of virtual patients *n*_*s*_ for each group is selected according to the weights in Figures 5, 6.

Once the optimal number of added virtual patients for each group, $n_{s}^{\text{R}}=26$ and $n_{s}^{\text{C}}=25$, is decided, our modelling approach enables the evaluation of the end point in an augmented clinical trial. The posterior distributions of the odds within each group from the *in vivo* and augmented trials are shown in Figure 7. As displayed on the top panel of the figure, 36% of the patients have negative sputum culture for the Control whereas 41% for the RUTI group. The posterior expected odds of recovery from each trial in the Control group remain quite similar, with a slightly wider equally tailed interval of probability 0.95 from the augmented trial. The addition of patients to the RUTI group does not seem to have a significant impact on inference, with both the posterior mean and 95% interval shifting slightly to the left; numerical summaries can be found in Table 3.

**Table 3:** Summary statistics (2.5, 50, and 97.5% quantiles) and IQR of the posterior odds of the Control and RUTI groups from the *in vivo* (*n*_*v*_) and augmented trials after adding the optimal size batch of virtual patients, $\left(n_{s}^{o}\right)$.

**Group**	**Model**	***n*_*v*_**	** $n_{s}^{o}$ **	***q*_0.025_**	***q*_0.5_**	***q*_0.975_**	**IQR**
Control	*In vivo* only	75	0	0.328	0.538	0.863	0.182
	augmented		25	0.331	0.544	0.876	0.184
RUTI	*In vivo* only	75	0	0.384	0.651	1.073	0.229
	augmented		26	0.370	0.634	1.059	0.229
Ratio	*In vivo* only	75	0	0.592	1.210	2.456	0.592
	augmented		25 + 26	0.565	1.162	2.355	0.580

**Figure 8:**
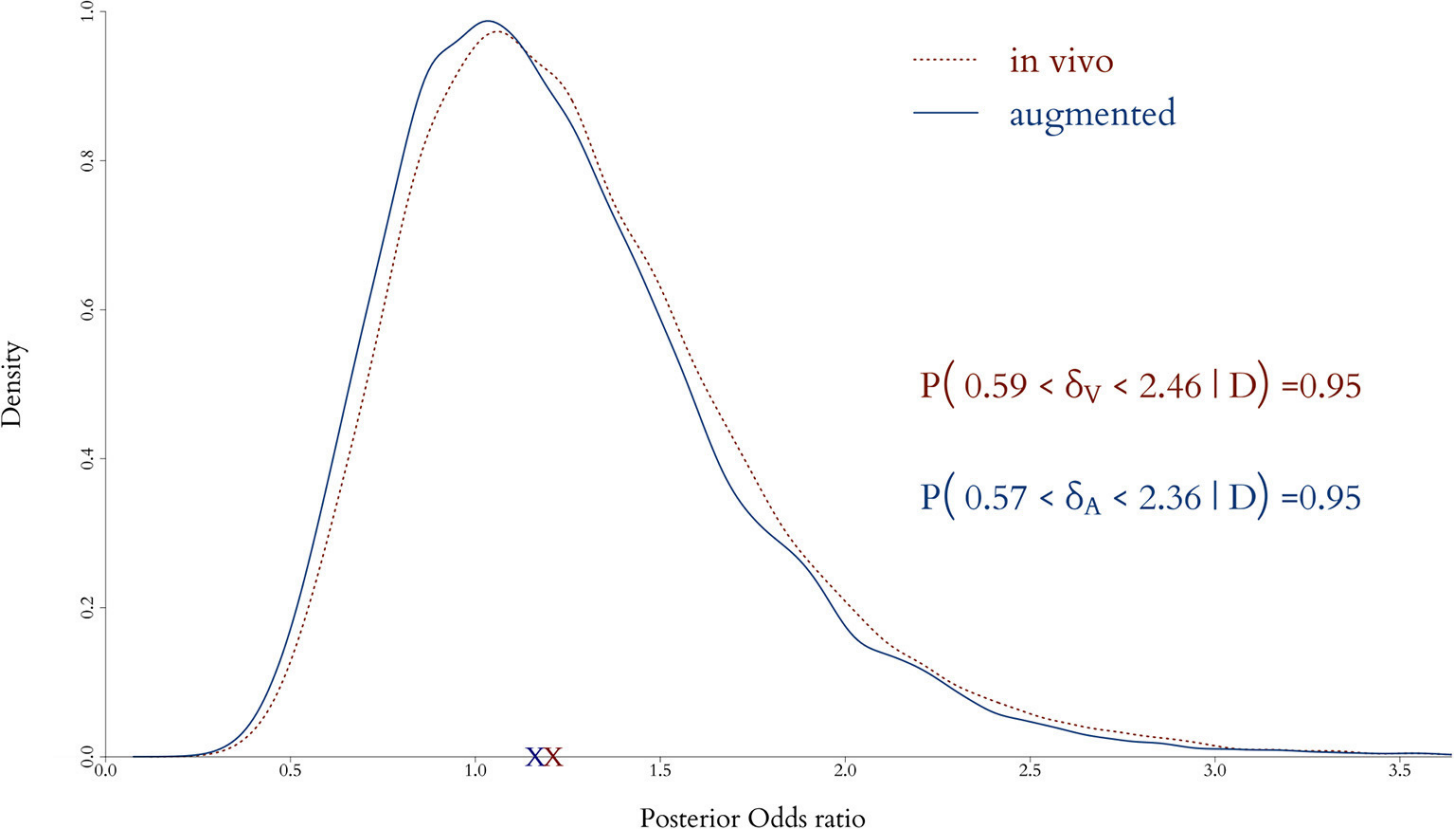
Posterior distributions of odds ratio when only the *in vivo* cohort is fitted and when both the *in vivo* and *in silico* cohorts are combined for the full model. Adding virtual patients does not alter significantly in the posterior expected odds but provides a decrease in the uncertainty in the estimation of the clinical end point.

The effect of augmentation to the end point is shown in Figure 8, where the posterior distributions of the odds ratio from the *in vivo* and augmented clinical trials are plot together. The red dotted line represents the *in vivo* only and the solid blue line the augmented trial, along with the posterior means (crosses) and equally tailed 0.95 posterior probability intervals. It is apparent that posterior point estimates are quite similar and would lead to the same clinical conclusion, but the width of the posterior interval of the augmented trial is shorter, providing increased precision.

Our models have been built in RStan (29), which enables straightforward implementation and modification of the prior. The code is available from our GitHub repository^3^ under request. On average, running our code takes a few minutes for 100s and slightly longer for 1,000s of patients, the individual models with the random effect—i.e., the *in silico* patients—being the slower. For instance, 750 *in vivo* patients with 500 *in silico* patients take a couple of minutes for both independent and combined models, whereas 3,750 *in vivo* with 2,500 *in silico* patients runs in about 25 min for the individual model with random effects and in around 10 min for the combined. We should point out that, despite not suffering from scaling issues, our models have been devised for clinical trials with limited number of *in vivo* patients, augmented with computer simulations to boost the performance with respect to the endpoint of the trial.

## 4. Discussion

The need for innovative solutions for improving public health is increasingly apparent. Speeding up the delivery of interventions to the market, without compromising safety and effectiveness, can be achieved by adding relevant information to the either clinical trial phase. Our methodology is designed to incorporate relevant information from *in silico* experiments onto clinical trials data to decrease their size and duration. There are indeed a number of approaches to simulate different aspects of TB, from purely synthetic approaches for designing new multi-epitope subunit vaccine (30) and molecular dynamics (31), to contagion dynamics (32). There are also models able to simulate pharmacokinetics in TB on a population level (33, 34), but none of these produce data relevant to the endpoint of the clinical trial considered in this study. UISS-TB is a bespoke ABM capable of simulating cohorts of TB *in silico* patients treated with RUTI (12, 24), which has been through ASME V&V 40-2018 (23); to the extent of our knowledge, currently there is no available alternative.

We illustrate our approach within an augmented Phase II clinical trial of a co-adjuvant vaccine for treating patients with TB. Both sources of information, *in vivo* and *in silico*, are combined using a novel statistical coherent Bayesian approach capable of propagating the uncertainty from both sources of information onto the posterior distribution of the clinical endpoint. The contribution of the *in silico* experiment is controlled by a measure of compatibility with the *in vivo* data and weighted accordingly into the combined trial. The models for the each source of information are tailored to the clinical endpoint, in our case study a GLM due to the binary endpoint, but alternatives can be readily used while adapting the prior structure accordingly.

We use a novel, bespoke simulator for the RUTI clinical trial, but the methodology for sharing information can be applied in a variety of scenarios. In principle, *in silico* data could be acquired from a number of sources (statistical, machine learning, and artificial intelligence) if relevant, adding the corresponding terms to Equation (2) to account for their different nature. In any case, appropriate choice of the penalty function is key, as it controls the quantity of information from the *in silico* experiments. This information balance must be considered carefully and may be indeed effected by a regulator by setting the maximum number of virtual patients, *m*_max_. We adapt the approach on Haddad et al. (25) and heuristically set its parameters by inspecting their discrimination power. A potential avenue for future research would be to provide a formal approach for automatic selection, enabling an unsupervised method for deciding the optimal number of virtual patients. Our penalty function is bounded in (0, 1) facilitating weighing *in silico* information regardless of the underlying distributions or sample sizes. This penalty could also be based on alternative measures of discrepancy between the two endpoint distributions, e.g., Kullback-Leibler, Chernoff, *L*_*m*_-norm (35), but would require scaling on case-by-case basis. Exploring alternative specifications for the penalty function and comparing relative performances are active part of our research plans.

By incorporating new virtual patients sequentially and re-evaluating their contribution to the augmented trial, the method enables sharing information efficiently without overwhelming the information from *in vivo* trial and thus improving the precision in the evaluation of the clinical endpoint without biassing the clinical decision. We hypothesise there are large gains to be obtained by using this kind of technology on well-explored diseases, where computer simulations are ripe for use and clinical trials common. For uncommon or rare diseases, this approach offers the possibility to improve on exploratory analyses where costs of recruitment and follow-up are large or even prohibitive.

## Data Availability Statement

The original contributions presented in the study are included in the article/supplementary material, further inquiries can be directed to the corresponding author/s.

## Author Contributions

DK and MJ developed the Bayesian methodology and carried out the analysis and drafted the manuscript. GR, GS, and FP developed the simulator. All authors participated in the generation of virtual patients, read, and approved the draft.

## Funding

The authors acknowledge support from the STriTuVaD project, funded by the European Commission under the contract H2020-SC1-2017- CNECT-2, No. 777123.

## Author Disclaimer

The information and views set out in this article are those of the authors and do not necessarily reflect the official opinion of the European Commission. Neither the European Commission institutions and bodies nor any person acting on their behalf may be held responsible for the use, which may be made of the information contained therein.

## Conflict of Interest

The authors declare that the research was conducted in the absence of any commercial or financial relationships that could be construed as a potential conflict of interest.

## Publisher's Note

All claims expressed in this article are solely those of the authors and do not necessarily represent those of their affiliated organizations, or those of the publisher, the editors and the reviewers. Any product that may be evaluated in this article, or claim that may be made by its manufacturer, is not guaranteed or endorsed by the publisher.

## Footnotes

^1^https://www.euro.who.int/en/health-topics/communicable-diseases/tuberculosis [accessed August 23, 2021].

^2^*In silico* trials for tuberculosis vaccine development. Available at: https://www.strituvad.eu/ [accessed August 23, 2021].

^3^https://github.com/kiagiasdim/AugTrial_UISSTB.git.
